# Biographical Sketch of the Late Dr. John O’Reilly of New York

**Published:** 1869-02

**Authors:** 


### ART. III.—*Biographical Sketch of the late Dr. John O'Reilly of New York.*

In the death of Dr. JOHN O'REILLY, the subject of this memoir, his professional brethren have to deplore the loss of a member of extraordinary devotion to the honor and best interest of their profession, and the general advancement of medical science.

John O'Reilly, the son of Gerald O'Reilly, was born at Ballinlough, in the county of Meath, Ireland, in the year 1813. From his earliest years he seems to have responded to his vocation; and both from his natural aptness to the pursuits, and the stimulating example of older members of his family, who distinguished themselves in the same profession; he made every other object secondary to the great end of his ambition, the attainment of high excellence as a surgeon and medical doctor.

After a diligent pursuit of classical study, he entered the Royal College of Surgeons in Ireland, (Dublin,) in 1831, in his 18th year, and for the next eight years of his life he unremittingly devoted himself to the study of surgery and medicine, attending regular courses of lectures on every branch of the profession. He had certificates of attendance for three years at the Richmond Hospital alone, and at the Whitworth Hospital he also spent two years, one as a dresser, and the other as clinical clerk. He then entered the University of Glasgow, where he passed two years in the study of medicine. Finally, in 1839, he brought his studies to a close, having received a diploma as Licentiate of the Royal College of Surgeons in Ireland; a diploma from same college for Midwifery; a diploma from the Lying-in-Hospital, Great Britain street, Dublin; a diploma for Medical degree from Glasgow University; a diploma from the Ophthalmological Society, Glasgow; and subsequently, 1845, he got the degree of Fellow of the Royal College of Surgeons in Ireland.

Thus regularly qualified he settled down to practice in Oldcastle, the town in which he first went to school, and there he passed the first eleven years of his professional career with very great success—that is, as a practitioner. From the beginning and throughout his whole life, the doctor's great ambition was to prescribe for and heal the sick; every one that sought his aid was equally the object of his solicitude, in proportion to the gravity of his disease and the distress of his condition. Whether his patient was clothed in fine linen or in rags was a matter of total indifference to him—that he was clothed with humanity was all that he required. He was the idol of the poor as their doctor and as their friend. But this was not enough to make him prosperous. The neighborhood in which he practiced was, like most neighborhoods in Ireland, composed of two classes—the rich and the poor—between whom there was, if any, but slender sympathy; and so it happened that this very quality that endeared him to the people—his total indifference to the worldly rank of his patient—was of great prejudice to his advancement with the aristocracy. The consequence was that he never received their patronage. There were many instances in which they thwarted his charitable enterprises; a case for example was where he founded a dispensary at his own expense, in a very poor neighborhood, within the range of his practice; not one shilling of the subsidy, which the government usually contributed to such institutions, could he ever obtain.

He was, it is true, appointed to the Work House and Fever Hospital of his own town, during the terrible years of famine and disease in Ireland, from 1846 to 1849, when the medical officer in charge resigned his place as too laborious for the salary he received.

In 1849—the year of the great exodus from Ireland—the doctor with so many of his countrymen resolved to quit his native country and make the United States the land of his adoption. On his arrival in New York his success was immediate; he was well and favorable known in the profession in a short time, and in 1851 he was unanimously elected a member of the New York Academy of Medicine, on the introduction of Dr. Valentine Mott. From this time forward for fifteen years his career of usefulness in his profession was remarkable; the number of persons that sought his professional aid was immense. At first his practice was general, but he was soon compelled to abandon out-door attendance, except in cases of consultation and of important surgical operations; his time was principally given to patients in his office, where he introduced a plan for the despatch of business, somewhat novel in this country, partly suggested by the custom of distinguished physicians of London and Dublin, and partly by the circumstances of his case. In summer time he was in his office at six in the morning, and in winter as soon as daylight; there he continued up to eleven. He never spoke about fees; this he left to his attendants; he kept no accounts. After the first visit, if his patient needed further treatment, and from his circumstances he was unable to pay, he had a free pass to come as often as necessary until his case was satisfactorily disposed of. Twice a week (Tuesdays and Fridays) his time was totally given to the poor, to whom he gave the price of the necessary medicines. From two in the afternoon he was again in his office, where he remained till five o'clock. On the first visit every patient seriously affected was thoroughly examined, and his case so thoroughly diagnosed that no one ever left him unconvinced that he exactly knew the nature of his disease. To his great natural ability and experience in this respect was due the great confidence which he never failed to inspire into his patient's mind. He rarely saw fewer than fifty patients each day, and the usual number was from seventy to eighty. He was called among his *clientel* "the surgeon," but he was equally good as a physician. Every new remedy that offered in the journals of the day, either in Europe or America, he sought a favorable opportunity to test. But in grave cases he confined himself to those remedies, the effect of which he exactly and undoubtedly knew, and on which he could implicitly rely.

Notwithstanding the amount of duty the doctor thus performed he made time for study; the number of lesser articles which he wrote on the remarkable cases that came under his treatment as surgeon and physician, would, if collected, fill several volumes. About the year 1860 he commenced to give particular study to the subjects of the Placenta and the Nervous Systems, and his anxiety to see them thoroughly investigated by the ablest minds in the profession was very great. Upon this subject he wrote two or three books; but he was by no means satisfied with all he did, and in order to attract the attention of the profession more directly to the point, he offered in 1866, through the Academy of Medicine, a prize of $600 for the best essay on the subject. But this he had not the satisfaction of seeing distributed in his time. For those labors and his writings generally the Royal Academy of Belgium elected him one of their correspondents in 1867.

But the doctor fairly overworked himself, and the consequence was that in January, 1865, his right side was completely paralyzed; he was never the same again; no more than the oak that the lightning has smitten in the forest, did he regain his native vigor. 'Tis true he rapidly regained the use of his limbs, and under the able and brotherly care of his friend, Dr. Nelson, who watched over him untiringly, he grew remarkably well, all things considered, and continued so for a year and a half. From that time forward he was afflicted with slight attacks, at first rare in their occurrence, but afterwards they became more frequent and severe to the close. But his hope never forsook him, the energy of his mind buoyed him up to the last, and in a wonderful way prevailed against the disease. Up to the tenth day before his death he continued to see a limited number of patients, and he prescribed for them with as much effect as ever. And it was only on that day that he fairly gave up. He desired to retire to bed about midday, and as he ascended he remarked that "*it was the last time.*" From this day he sank rapidly, and on the sixth of December, 1868, he expired.

So ended the life of Dr. John O'Reilly, at the age of 56. He was a man decidedly remarkable in his profession, for his great success in the treatment of diseases, and the performance of surgical operations. In this latter respect his *good luck* (as he called it) was unprecedented. In all his important operations his patient invariably recovered, for he never operated unless he was convinced that thereby he would confer a permanent benefit. The operation for *hernia* was one he very often performed; his plan in this case—as he wrote it in one of his articles—was to lose no time; he made one vigorous effort to reduce the hernia by *taxis*, and if that failed he at once resorted to the knife. His operations of *lithotomy* numbered eight—all successful. In operating he was very rapid; never was there an instant of hesitation or doubt. He operated for cataract four times. He never operated in this case unless the patient was completely blind.

In his diagnosis he was also quick; as if by instinct he almost divined his patient's constitution, age, calling, nay, almost his habits, without being told.

His sympathy for his unfortunate patient was very great. He always thought of them, and with such intensity that his family could tell when he had a serious case in charge. With him money was always a secondary consideration; *he never in all his life demanded a fee.* His private charities he kept profoundly secret; no one would ever have known of them if he carried his own purse. What he did of a public nature he told to everybody. There was absolutely no affectation or conventionality about him. In his family he was childlike; he loved children, and pets of every kind he always kept about him. He was very industrious, always reading or writing. He was of a most amiable character; he really never by word or act injured any one, and his memory no doubt is gratefully enshrined in the hearts of the thousands whom he benefited. *Requiescat in pace.*

---

A YOUNG MOTHER.—Our correspondent, Dr. King, of Rochford, Essex, has forwarded us a communication in which he states that he had recently attended the confinement of a girl under eleven years of age. The mother and infant were both well. Dr. King verified the fact by an inspection of the girl's register of birth. This is probably the youngest example on record, and we earnestly hope that it may continue to be so, for it manifests a depraved precocity which is truly lamentable in a Christian country.—*Lancet.*

